# Positron Emission Tomography-Computed Tomography and Magnetic Resonance Imaging Assessments in a Mouse Model of Implant-Related Bone and Joint Staphylococcus aureus Infection

**DOI:** 10.1128/spectrum.04540-22

**Published:** 2023-04-03

**Authors:** J. J. Aguilera-Correa, B. Salinas, M. González-Arjona, D. de Pablo, P. Muñoz, E. Bouza, M. J. Fernández Aceñero, J. Esteban, M. Desco, L. Cussó

**Affiliations:** a Departamento de Química en Ciencias Farmacéuticas. Universidad Complutense de Madrid, Madrid, Spain; b CIBERINFEC-CIBER de Enfermedades Infecciosas, Instituto de Salud Carlos III, Madrid, Spain; c Unidad de Medicina y Cirugía Experimenta, Instituto de Investigación Sanitaria Gregorio Marañón, Madrid, Spain; d Departamento de Bioingeniería, Universidad Carlos III de Madrid, Madrid, Spain; e Unidad de Imagen Avanzada, Centro Nacional de Investigaciones Cardiovasculares, Madrid, Spain; f CIBER de Salud Mental, Instituto de Salud Carlos III. Madrid, Spain; g Servicio de Anatomía Patológica Hospital Clínico San Carlos, Fundación para la Investigación Biomédica HCSC, Madrid, Spain; h Servicio de Microbiología y Enfermedades Infecciosas, Hospital General Universitario Gregorio Marañón, Madrid, Spain; i Departamento de Medicina, Facultad de Medicina, Universidad Complutense de Madrid, Madrid, Spain; j CIBER Enfermedades Respiratorias, CIBERES, Madrid, Spain; k Clinical Microbiology Department, IIS-Fundacion Jimenez Diaz, UAM, Madrid, Spain; Riverside University Health System, Medical Center, University of California

**Keywords:** *Staphylococcus aureus*, osteoarthritis, implant-related infection, molecular imaging

## Abstract

Osteomyelitis is an infection of the bone, associated with an inflammatory process. Imaging plays an important role in establishing the diagnosis and the most appropriate patient management. However, data are lacking regarding the use of preclinical molecular imaging techniques to assess osteomyelitis progression in experimental models. This study aimed to compare structural and molecular imaging to assess disease progression in a mouse model of implant-related bone and joint infections caused by Staphylococcus aureus. In SWISS mice, the right femur was implanted with a resorbable filament impregnated with S. aureus (infected group, *n* = 10) or sterile culture medium (uninfected group, *n* = 6). Eight animals (5 infected, 3 uninfected) were analyzed with magnetic resonance imaging (MRI) at 1, 2, and 3 weeks postintervention, and 8 mice were analyzed with [^18^F]fluorodeoxyglucose (FDG)-positron emission tomography (PET)-computed tomography (CT) at 48 h and at 1, 2, and 3 weeks postintervention. In infected animals, CT showed bone lesion progression, mainly in the distal epiphysis, although some uninfected animals presented evident bone sequestra at 3 weeks. MRI showed a lesion in the articular area that persisted for 3 weeks in infected animals. This lesion was smaller and less evident in the uninfected group. At 48 h postintervention, FDG-PET showed higher joint uptake in the infected group than in the uninfected group (*P* = 0.025). Over time, the difference between groups increased. These results indicate that FDG-PET imaging was much more sensitive than MRI and CT for differentiating between infection and inflammation at early stages. FDG-PET clearly distinguished between infection and postsurgical bone healing (in uninfected animals) from 48 h to 3 weeks after implantation.

**IMPORTANCE** Our results encourage future investigations on the utility of the model for testing different therapeutic procedures for osteomyelitis.

## INTRODUCTION

The overall incidence of osteomyelitis is currently estimated at 21.8 cases per 100,000 person-years. The incidence is relatively similar between children and young adults, but it is much higher in individuals over 60 years old, probably due to the prevalence of comorbidities, such as diabetes mellitus and peripheral vascular disease (1, 2).

Over 80% of osteomyelitis cases are caused by Staphylococcus aureus infections (3). Furthermore, most (and probably all) of the microorganisms that cause osteomyelitis can develop a biofilm, a growth form that allows bacterial survival in the presence of adverse conditions, such as immune system phagocytosis or antimicrobial administration (4). Due to the presence of biofilm, the treatment for osteomyelitis (particularly chronic forms) typically involves the surgical removal of infected bone or devices, followed by prolonged antibiotic therapy (5).

Imaging is an essential tool for both diagnosing osteomyelitis and determining its treatment and follow-up (6). The most common imaging techniques include magnetic resonance imaging (MRI) and computed tomography (CT) (7). However, MRI is significantly limited after metallic material implantations, because metals generate severe imaging artifacts (8) that prevent the use of MRI in the context of bone-related infections. In these cases, conventional radiography (9) and CT (7) are common alternatives, although they may yield false-negative results and underestimate the true extent of the infection (9). Moreover, they may not have sufficient sensitivity for early detection (7).

Structural imaging can be complemented or replaced by nuclear medicine techniques, such as positron emission tomography (PET), single positron emission computed tomography (SPECT), and scintigraphy (7). Studies have demonstrated the usefulness of PET with [^18^F]fluorodeoxyglucose (FDG) for diagnosing several types of S. aureus infections, such as bacteremia (10). Nevertheless, little information is available on the value of PET in the diagnosis and follow up of human bone infections.

Animal models can be useful, and many mouse models have gained importance (11, 12) in preclinical settings. However, the sensitivity of structural imaging techniques depends heavily on the animal model (13). Horst et al. (14) showed that MRI and CT could reveal bone structure changes during the chronic stage (i.e., 1 month after bacterial inoculation) of hematogenous osteomyelitis caused by S. aureus in a mouse model. However, those techniques lacked early detection sensitivity. Conversely, Li et al. (15) reported that CT could detect evident bone damage, starting on day 7, in a murine model of tibial implant-associated osteomyelitis, caused by a steel pin coated with S. aureus. PET studies of rabbit bone and joint infections showed that FDG-PET could distinguish between infected and uninfected tissues (16–18) and even between postsurgical bone healing and infection at 3 weeks after implantation (17). However, we lack data on the relative usefulness of the different imaging modalities regarding the longitudinal progression of osteomyelitis in mouse models.

The present study aimed to compare structural (CT and MRI) and molecular (FDG-PET) imaging to assess disease progression in a mouse model of implant-related bone and joint infections caused by S. aureus.

## RESULTS

### Model validation and clinical results.

Body weight reductions were observed at 24 h postintervention in both groups (Fig. S1), and weight loss continued at 48 h postintervention (*P* < 0.05, with respect to the baseline). The uninfected group recovered to baseline weight at 72 h. In contrast, the infected group did not completely recover for almost 10 days. Weight changes were significant on days 2 (*P* = 0.012), 3 (*P* = 0.009), and 9 (*P* = 0.004) postintervention compared to day 0. However, body weights were not significantly different between the two groups.

Among the clinical signs, both groups developed lameness. In the infected group, 60% of animals developed lameness at 24 h after the intervention, and this proportion increased over time (80% of animals were lame on day 16). In the uninfected group, 50% of animals developed lameness at 24 h, and this proportion decreased to 17% on day 16. In addition, 30% of infected animals showed piloerection at 24 h, and this clinical sign persisted over time in approximately 40% of the animals. In contrast, piloerection occurred only occasionally in the uninfected group (see Table S1 in the supplemental material). Neither group exhibited lack of grooming, wounds, passivity, aggressiveness, or mortality.

### Pathology and microbiology.

**FIG 1:**
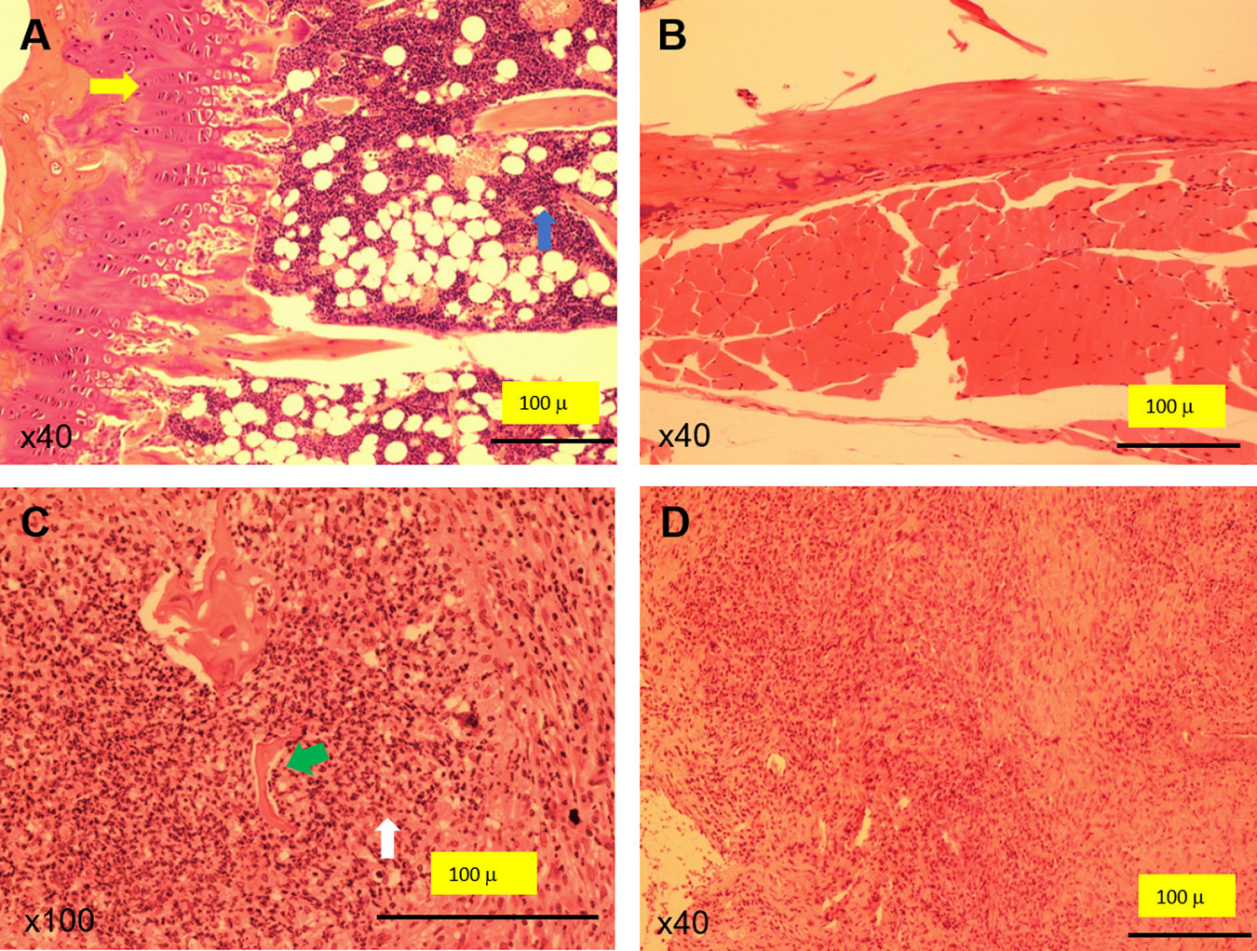
H&E staining. Micrographs show representative tissue samples from uninfected (A and B) and infected (C and D) mice. (A) Normal joint with preserved cartilaginous plate (yellow arrow) and marrow-rich cancellous bone (blue arrow). Note the abundant hematopoietic cells. (B) Normal appearance of skeletal muscle in the soft tissues surrounding the joint. Noninflammatory infiltrates are observed. (C) Severe inflammation involving the bone. Note the prominence of osteoblasts in the bone trabeculae (green arrow), showing irregular edges. Polymorphonuclear neutrophils are also evident (white arrow). (D) Inflammatory infiltrates in the soft tissues surrounding the joint. Magnifications, ×40 (A, B, and D) and ×100 (C).

**FIG 2:**
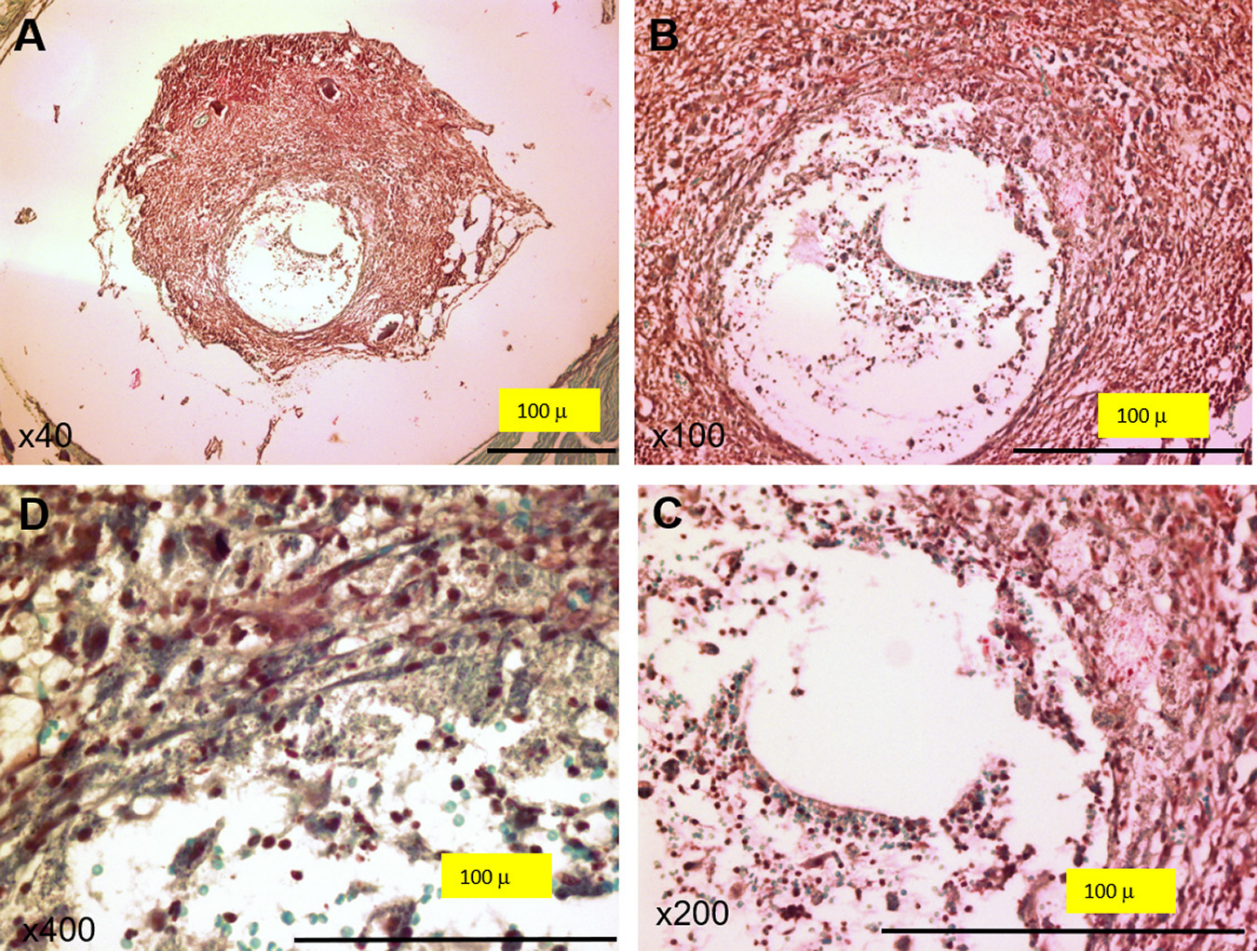
Gram-stained tissue samples. Representative tissue sample from an infected mouse. (A) Low-power microscopic view showing the implant site surrounded by dense inflammatory infiltrate, mainly consisting of (B) polymorphous leukocytes; (C) the implant (empty space) surrounded by inflammatory infiltrates; (D) Gram-positive organisms. Magnifications, ×40 (A), ×100 (B), ×200 (C) and ×400 (D).

Histological data (Table 1 and Fig. 1) showed that 100% of infected animals developed inflammation in the joint and bone. Of 5 animals, 2 developed inflammation in the periarticular soft tissues and 4 d0eveloped inflammation in the muscle. The uninfected group showed no signs of inflammation in any of the tissues studied. Gram staining of the paraffin-embedded tissues confirmed the presence of Gram-positive bacteria in the infected animals (Fig. 2). Additionally, a microbiological analysis (Table 1) of the femur confirmed the presence of S. aureus in all femurs of mice with infected implants. No bacterial growth was observed in the uninfected group.

**TABLE 1:** Histological and microbiological results for tissues from infected (*n* = 5) and uninfected (*n* = 3) mice

Group and mouse	Histological score for^*a*^:				Log_10_(CFU of bacteria/g)
	Muscle	Periarticular soft tissue	Bone	Joint	
Uninfected					
1	0	0	0	0	0
2	0	0	0	0	0
3	0	0	0	0	0
Infected					
4	2	0	2	2	5.329
5	2	2	2	2	6.005
6	2	1	2	2	5.032
7	0	0	2	2	4.572
8	2	0	2	2	5.080

a 0, absence of inflammation; 1, mild inflammation; 2, severe inflammation (abscess).

### Computed tomography.

**FIG 3:**
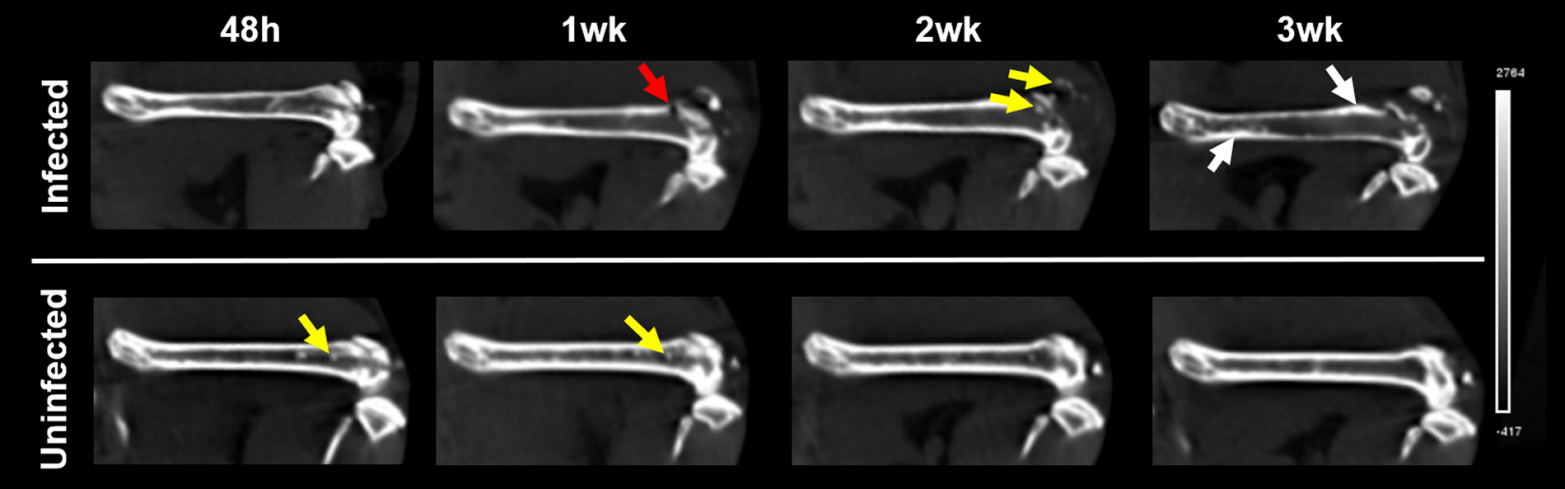
CT images of femurs. Representative images of femurs and knee joints in infected (top) and uninfected (bottom) animals over time. At 48 h, the filament access points were clearly visible in the distal epiphysis in all animals. Bone infection progressed over time and mainly affected the distal epiphysis. These representative images show sequestra (yellow arrows), abscesses (red arrow), and involucres (white arrow).

At 48 h, in both groups, the CT study showed posttraumatic bone fragments in the femoral medulla, and the filament access point was visible in the distal epiphysis in all animals (Fig. 3). One week after the intervention, all infected animals presented an enlargement at the filament access point, accompanied by edema in the soft tissues and medullary, and sequestra in the distal epiphyseal bone (Fig. 3). In contrast, two of the three uninfected animals presented evident bone sequestra in the medullary canal, which disappeared over time. At 3 weeks, sequestra persisted only in one uninfected animal. In the infected group, bone infections progressed over time, mainly affecting the distal epiphysis, which showed several pathological features (sequestra, abscesses, and involucres) suggestive of osteonecrosis. The CT lesions in the distal epiphysis were consistent with the areas of high FDG uptake.

### Magnetic resonance imaging.

**FIG 4:**
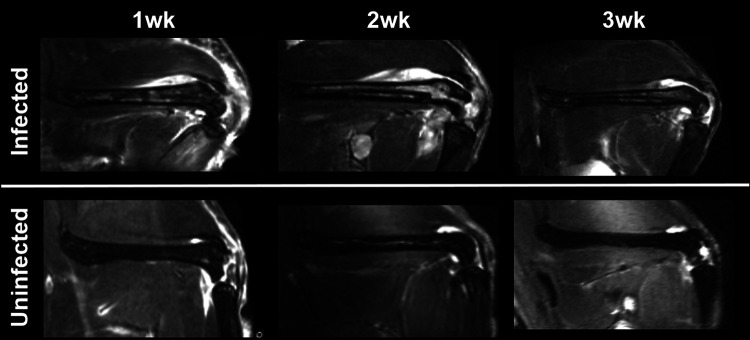
Representative MR images of femurs in infected and uninfected animals over time. (Top) Infected animals showed persistent edema in the soft tissue around the joint and distal diaphysis over time. (Bottom) The extent of edema is clearly lower in uninfected animals.

The MRI study (Fig. 4) showed that the infected group developed a lesion in the joint area, which persisted over time. This damage was smaller and less evident in the uninfected group. At the medullary level, we did not observe any differences over time or between the groups.

### FDG-PET.

**FIG 5:**
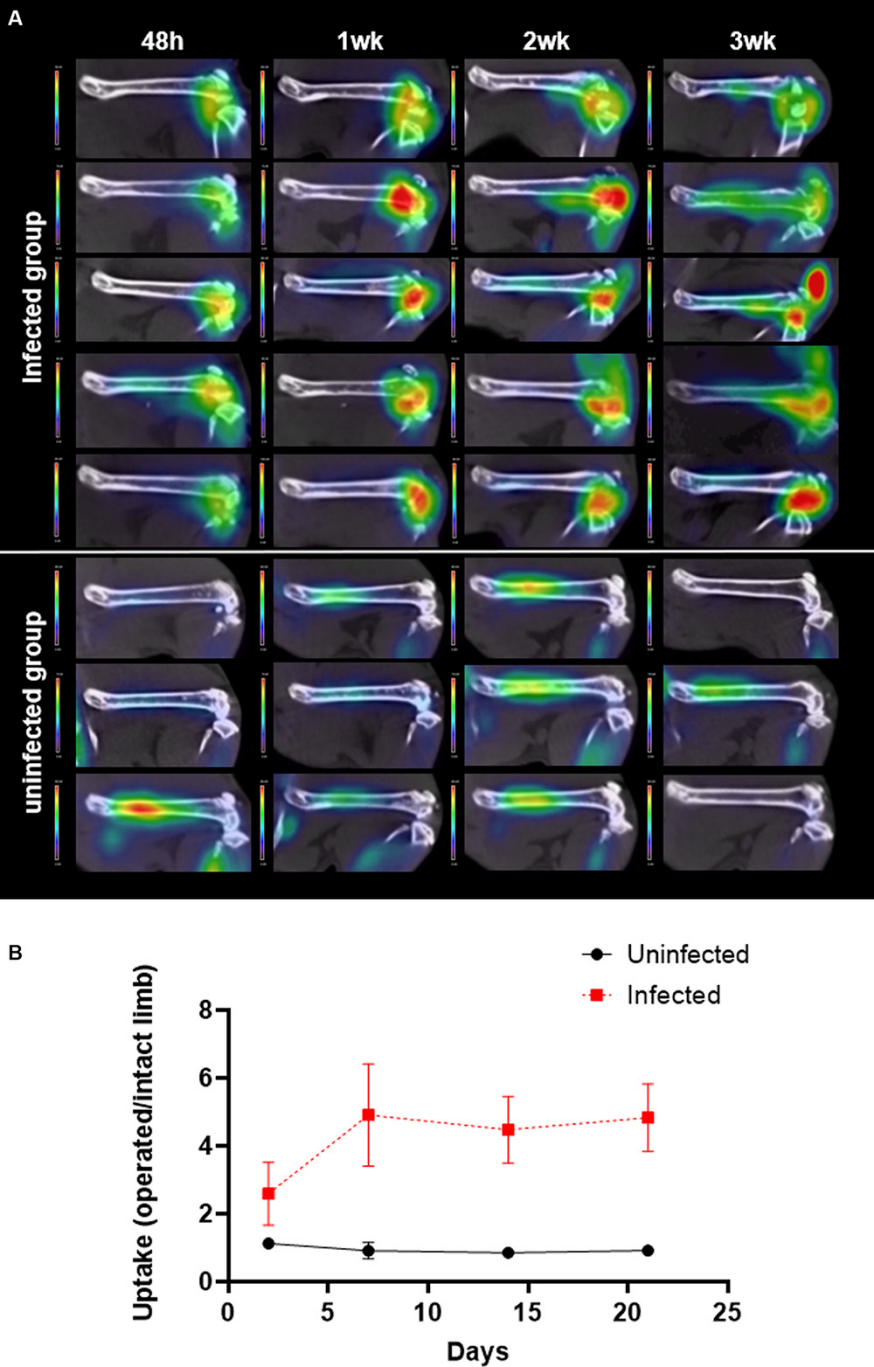
Time course of FDG uptake in femur and joint after implantation. (A) FDG-PET-CT images show FDG uptake (color bar in SUV units). (B) FDG uptake is quantified as the SUVmean ratio between the limbs that were operated on and the intact limbs. *P* < 0.05 in the infected group compared to the 48-h SUVmean.

**FIG 6:**
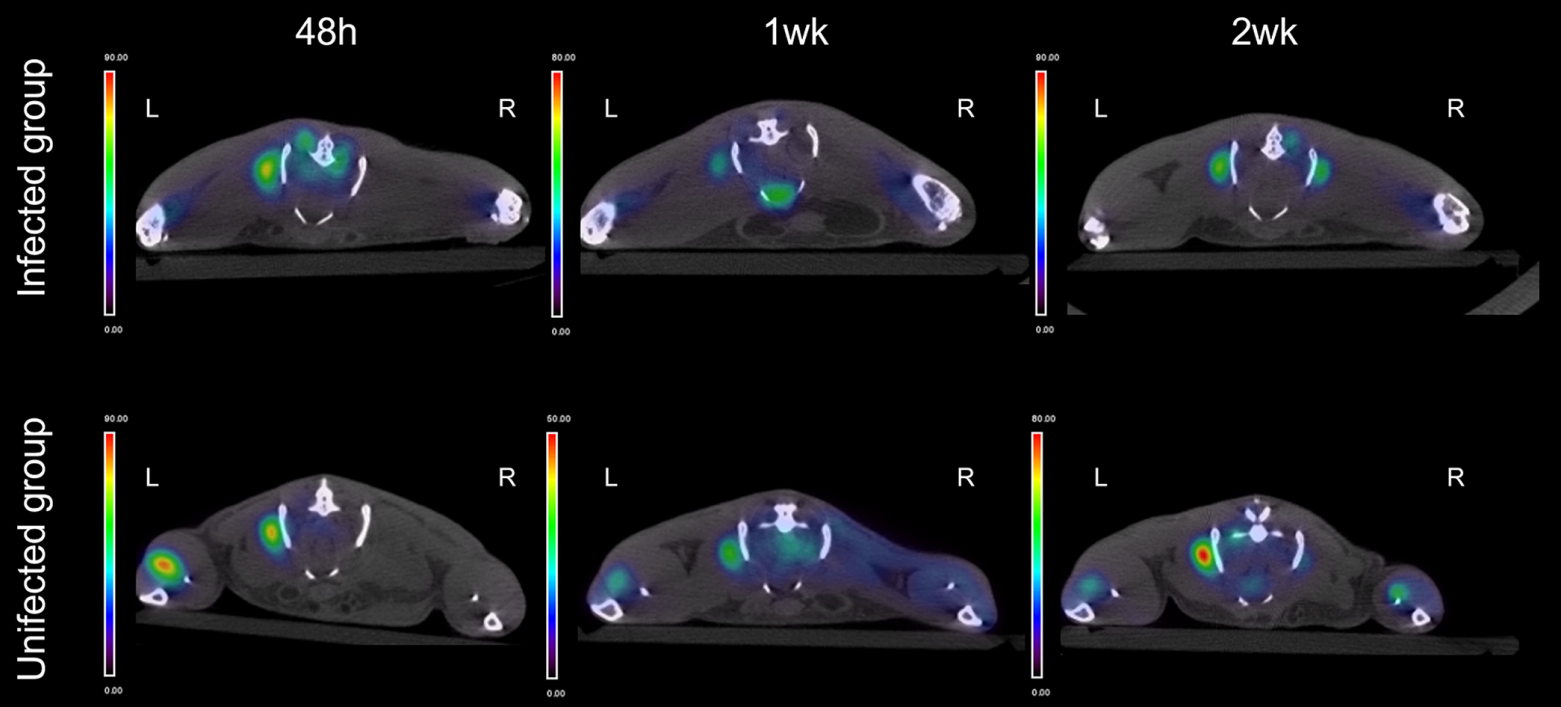
Time course of muscle FDG uptake after implantation. Axial views of representative FDG-PET-CT images of infected (top) and uninfected (bottom) mice. FDG uptake (color bar in SUV units) was increased on the intact limb (left sides [L]) compared to the limb that was operated on (right sides [R]).

Figure 5A shows the FDG-PET-CT images of the limb that was operated on in all animals. At 24 h after the intervention, the infected group showed significantly higher FDG uptake in the joint than the uninfected group (*P* = 0.025). Moreover, the difference between groups increased to 5-fold after 1 week. Over time, no significant changes were observed in the uninfected group (*P* > 0.05). In the infected animals, FDG uptake nearly doubled at 1 week (*P* = 0.043 compared to the 48-h uptake) and remained elevated (Fig. 5B). Conversely, we observed an elevated FDG uptake in the intact limb musculature (e.g., the gluteus, paravertebral muscles, and anterior tibialis) of all animals at 48 h, compared to the limb that was operated on, which remained stable over time (Fig. 6).

## DISCUSSION

This study demonstrated that FDG-PET imaging is more sensitive than CT and MRI in differentiating between infection and inflammation in a successful mouse model of implant-related bone and joint infection.

Bone infection is a complex process, and it is difficult to make a differential diagnosis between infection and noninfective conditions. The diagnosis and follow-up of bone infections with imaging techniques are essential for determining the adequate treatment and an appropriate treatment duration (6).

CT and MRI are the most common imaging modalities for use in humans (6). However, both these techniques have significant limitations, in terms of initial diagnostic sensitivity, partly due to the artifacts derived from the presence of prosthetic material. In addition, neither technique can always differentiate between infection and sterile inflammation. Nevertheless, animal models of bacterial bone infection have proved to be essential in translational research (13, 19). In the present study, we used a radiolucent resorbable material to create a focus of infection that mimicked osteomyelitis. The use of this material improved the image quality, compared to metallic implants, because it prevented the appearance of CT and MR imaging artifacts (20).

According to a review by Guarch-Pérez et al. on mouse studies (11), in 59% of studies, infection progression was evaluated by sacrificing the animals (at each time point) to measure the CFU. Moreover, the use of imaging techniques in osteomyelitis models was limited; the most common modalities were CT (40%, including *in vivo* and *ex vivo* studies), radiography (20%), and MRI (<5%). Those authors also highlighted the use of bioluminescence imaging, although they did not quantify the results. To our surprise, there was no mention of or data on nuclear imaging.

Consistent with previous studies (21), the animals in both our groups showed signs of pain (limping and piloerection). Although a lower proportion of uninfected animals than infected animals were affected, and although uninfected animals recovered over time, the differences between groups hardly reached statistical significance. We also observed body weight reductions at 24 h postintervention in both groups. This weight loss was recovered more rapidly in the uninfected group than in the infected group (72 h versus 10 days), but the difference was not statistically significant. Therefore, clinical variables did not offer statistically reliable information for distinguishing between infected and uninfected animals, even though these variables are commonly reported in animal models of joint bone infection (14, 18, 21). Our histopathological results showed that our model is associated with a more intense leukocytic inflammatory response than is usual in human samples. These differences may be due to the lack of human tissue samples from acute phases of osteomyelitis, which is rarely, if ever, biopsied for histopathological analysis, but also due to the high bacterial dose in mice, which often gives rise to rapid lysis of bacteria by complement (22), which might induce an intense leukocytic inflammatory response (23).

In our study, MRI results did not provide relevant information about the progression of the infection or signs for distinguishing between infected bone and postoperative bone healing (uninfected group). These results highlighted the limitations of the MRI technique for detecting small lesions. In our model, inflammatory lesions in soft tissues were observed with MRI at 1 week, and they were slightly more pronounced in the infected group, in agreement with previous studies (14). However, our model did not show clear medullary inflammatory lesions (hyperintense areas), as described by Horst et al. (14).

With CT imaging, bone damage was evident in both groups at 7 days after the intervention, although bone destruction progressed only in infected animals. This timing for detection was earlier than the one reported in previous studies (14). This earlier detection might be attributable to better scanner resolution or to the lack of metallic artifacts in our study, because we used resorbable material. Although our structural techniques were not quantitative, our results suggested that CT could be more sensitive than MRI for distinguishing between infection and bone healing, because as time progressed, CT detected damage reduction in uninfected animals over time; in fact, all these animals recovered almost completely at 3 weeks postintervention. In contrast, infected animals showed progressive bone infection over time. The infection mainly affected the distal epiphysis and showed several pathological features (i.e., sequestra, abscesses, and involucres) suggestive of osteonecrosis.

Our results showed that FDG uptake distinguished quantitively between infected bone and postoperative bone healing at 48 h after surgery, which was earlier than reported in previous studies (17). The difference between these conditions became more evident at 1 week and remained significant after 3 weeks, in agreement with previous studies (17, 18).

Nuclear imaging has been used in previous studies on bone infections (particularly with bone implants [24]), cardiovascular conditions, and fevers of unknown origin (7). FDG is the radiotracer most widely used due to its high sensitivity and availability. FDG-PET was reported to provide discrimination between aseptic and septic lesions and between postoperative infections and postoperative bone healing (25, 26). Moreover, FDG-PET showed superiority over structural techniques (20, 27). Nuclear imaging has become an indispensable tool in preclinical research on infections (24). It was used for evaluating new diagnostic radiotracers, such as [^18^F]fluorodeoxysorbitol or 1-(2′-deoxy-2′-fluoro-β-d-arabinofuranosyl)-5-iodouracil, for discriminating between bacteria and mammalian cells and for discriminating between different types of microorganisms (28).

Currently, there is general consensus about the relevance of combining nuclear imaging with structural techniques (CT or MRI). Some clinical studies on bone infection showed that combining PET-CT or SPECT-CT imaging with MRI had better diagnostic potential (29, 30). In our study, the combination of FDG-PET imaging with CT enabled a better anatomic localization of uptake. Full-body PET-CT scans also provided a means to observe other interesting patterns in our model, such as the FDG uptake increase in the musculature in the intact limb. This finding could be explained as a consequence of increased energy consumption in the musculature of the leg that was not operated on, due to the extra biomechanical load assumed when the infected leg (that was operated on) began limping. This finding exemplifies the advantages of this technique, particularly to detect unforeseen foci of increased uptake, such as infectious metastases (31). Finally, nuclear imaging was the only modality that enabled the acquisition of quantitative data. However, many studies of small animal imaging for inflammatory musculoskeletal conditions lack quantitative analysis (13). This feature constituted an advantage, because it enabled more objective, sensitive statistical assessments than purely structural techniques.

One of the main challenges in diagnosing bone infections is to distinguish between acute and chronic phases. A limitation of our design was that we did not address this challenge, because we did not study the histological evolution of these two phases in our model. Moreover, we did not attempt to coregister MRI and FDG-PET images, because that would require the use of a multimodal bed (32), which makes limb MRIs challenging. A blind analysis of the images was not performed, and the regions of interest (ROI) were drawn e over the area that qualitatively showed more differences between groups in order to reinforce the qualitative observations. Finally, we presented a descriptive study comparing imaging techniques and did not aim to obtain results of diagnostic accuracy, which would require increasing the number of animals.

In conclusion, this study objectively assessed with structural and molecular imaging the progression of implant-related bone and joint infections caused by S. aureus in a mouse model. We found that FDG-PET and CT imaging were more sensitive than MRI for diagnosing S. aureus infections in bone between 1 and 3 weeks after implantation. We found that FDG-PET was clearly superior to CT for distinguishing between infections and postsurgical bone healing (in uninfected animals) at 48 h after the implant placement. However, the PET-CT combination enabled more precise anatomical localization and, therefore, better FDG uptake quantification. Our mouse model clearly allowed early distinction between infection and inflammation with FDG-PET-CT imaging. These results encourage future investigations of the utility of the model for testing different therapeutic procedures for osteomyelitis.

## MATERIALS AND METHODS

### Experimental design.

This study included 16 11-week-old SWISS RjOrl:SWISS (CD1) mice (Janvier Labs, France). Four or five mice were housed per cage under a 12-h light-dark cycle at 23° ± 1°C and 50% ± 5% humidity. All mice were allowed access to food and water *ad libitum*. The animals were divided into infected (*n* = 10) and uninfected (*n* = 6) groups. Eight animals (5 infected and 3 uninfected) were analyzed with MRI imaging at 1, 2, and 3 weeks postintervention. Another 8 animals (5 infected and 3 uninfected) were analyzed longitudinally with FDG-PET at 48 h and at 1, 2, and 3 weeks postintervention.

### Implant preparation.

We used 1-cm resorbable implants (Biosyn 0; Synature) for infections and mock infections. For the infection group, each implant was deposited into a well of a 24-well cell culture plate (Thermo Fisher Scientific) and covered with 1 mL of a 1.5 MacFarland (~4.8 × 10^8^ CFU/mL) suspension of methicillin-susceptible S. aureus (Sa5) in saline (B. Braun). This clinical strain was used previously in an *in vivo* murine study (21). The plate was incubated for 2 h at 37°C and 5% CO_2_. After incubation, the supernatant was discarded, and the implant was ready for use. Uninfected implants covered with saline were used as controls.

### Animal surgical model.

The surgical intervention was based on a protocol previously described by Aguilera-Correa et al. (21). Briefly, the intervention consisted of placing a 1-cm resorbable implant into the right femur, through the knee, with an aseptic surgical technique. After the surgery, and during the entire experiment, the animals received 20 mg/mL of ibuprofen in the drinking water. Ten animals received implants infected with S. aureus, and six animals received sterile implants. Every other day, hamster food (Vital Menu; Vitakraft) was added to the usual food as an environmental enrichment measure. After surgery, the clinical condition of the animals was monitored up to day 21. We assessed the appearance of lameness, wounds, piloerection, lack of grooming, passivity, and aggressiveness, and we measured animal weight. Data are reported as percentages of baseline measurements.

### Model validation.

The animal model of implant-related bone and joint S. aureus infection was validated with histological and microbiological analyses. Animals were sacrificed after PET-CT studies, and the implanted femurs from 8 animals (5 infected and 3 uninfected) were removed. Each femur that had been operated on was divided into proximal epiphysis, distal epiphysis, and diaphysis, and the diaphysis was further divided into two sections. The distal epiphysis and the most proximal section of the diaphysis were used for histopathological analysis. The proximal epiphysis and distal diaphysis were used for microbiological analysis.

Briefly, the histological preparation consisted of sample fixation in 10% buffered formaldehyde for 24 h and subsequent decalcification in a Surgipath decalcifier II (Leica) for 12 h. Then, the samples were embedded in paraffin, cut into 3-μm sections, and stained with hematoxylin-eosin (H&E). A pathologist assessed the intensity of the inflammatory reaction in the muscle, periarticular soft tissue, bone, and joint for each sample, with the following semiquantitative scale: 0, no inflammation; 1, mild inflammation; and 2, severe inflammation. Gram staining (Artisan Gram stain kit; Agilent) was also performed on 3-μm sections of the samples.

For microbiological analysis, each sample was placed inside a sterile plastic bag and crushed with a hammer. The resulting crushed tissue was suspended in 2 mL 0.9% NaCl saline (B. Braun) and sonicated for 5 min at room temperature in a JP Selecta sonicator. The resulting liquid was seeded onto chocolate-blood agar (bioMérieux) via the spread plate method. For this method, samples were diluted 1:10, 1:100, and 1:1,000 in saline; then, 100 μL of the dilution was seeded onto an agar plate and spread with a Digralsky loop until completely absorbed. All plates were incubated at 37°C and 5% CO_2_ for 48 h. The final bacterial concentration was estimated by counting viable colonies, and the results are expressed in CFU per gram of sample.

### Imaging studies.

**(i) PET-CT studies.** Before the imaging studies, mice fasted for 8 h and were provided with water *ad libitum*. Lower-limb PET-CT studies were carried out with a SuperArgus small-animal scanner (Sedecal, Madrid, Spain). Briefly, mice were anesthetized with inhaled anesthesia (3% sevoflurane in 100% oxygen), and then 22.8 ± 0.85 MBq of FDG was administered by tail vein injection. After a 3-h uptake period (33, 34), animals were again anesthetized (3% sevoflurane in 100% oxygen) for PET-CT data acquisition (30 min). The respiration rate of the anesthetized animals was continuously monitored during the scan period with small-animal-dedicated equipment (VisionPet; RGB, Madrid, Spain). PET images were reconstructed with the OSEM-2D algorithm. The parameters were as follows: 16 subsets and 1 iteration; voxel size, 0.388 by 0.388 mm in the transaxial plane and 0.775 mm in the axial plane (system field of view [FOV], 67.8 by 67.8 by 47.3 mm) After the PET scan, a CT scan was performed with an X-ray beam current of 340 μA and a tube voltage of 40 kVp. We reconstructed the images with the Feldkamp, Davis, and Kres (FDK) algorithm (35).

**(ii) MRI studies.** Lower-limb MRIs were obtained with a 7 Tesla BioSpec 70/20 scanner (Bruker, Ettlingen, Germany) in anesthetized (3% sevoflurane in 100% oxygen) mice. A T2 coronal RARE sequence was acquired with a TR of 3511.64 ms, a TE of 30.02 ms, 2 averages, a rare factor of 8, and a slice thickness of 0.5 mm (35 slices). The matrix size was 256 by 256 pixels with a FOV of 25 by 42 mm.

**(iii) Data analysis.** PET-CT images were analyzed with Multimodality Workstation software (36) (MMWKS, Spain). On each CT image, cylindrical ROI (diameter, 2.0 mm) were drawn in the operated left joint and the intact right joint on 10 axial slices (16). These ROI were applied to automatically coregistered PET images to measure corresponding mean standard uptake values (SUVmean). SUVmean results are expressed as the ratio between the limb that was operated on and the intact limb. CT images were qualitatively analyzed by an expert. CT imaging findings were assessed as described previously by Lee et al. (37).

### Statistical analysis.

Repeated-measures analysis of variance (ANOVA) was performed to assess body weight variations within each group, with the surgery day weight (day 0) as the reference (simple contrast), and also to compare weight differences between groups. These data satisfied the assumptions of normality and homogeneity of variances. However, activity data did not comply with these assumptions. Therefore, we performed a Wilcoxon test to assess the progression of uptake in each group with the 48-h SUVmean ratio as a reference. We performed the Mann-Whitney U test to compare SUVmean ratios between groups at each time point. Data are reported as means (and standard deviations), and the statistical significance threshold was set at a *P* value of <0.05. The Fisher exact test was performed to analyze clinical signs (i.e., lameness, piloerection, lack of grooming, passivity, and aggressiveness).

### Ethics.

Mice were housed in the animal facility of the Hospital General Universitario Gregorio Marañón, Madrid (HGUGM), Spain (ES280790000087). All animal procedures conformed to EU Directive 2010/63EU and national regulations (RD 53/2013). All animal procedures were approved by the HGUGM Animal Experimentation Ethics Committee, the local Ethics Committees, and the Animal Protection Board of the Comunidad Autónoma de Madrid (PROEX 123.8/20).
