# Supplementary material for: Positron Emission Tomography-Computed Tomography and Magnetic Resonance Imaging Assessments in a Mouse Model of Implant-Related Bone and Joint Staphylococcus aureus Infection
Source: Microbiol Spectr. 2023 Apr 3;11(3):e04540-22. doi: 10.1128/spectrum.04540-22 (PMC10269916; doi:10.1128/spectrum.04540-22)
Supplement: Supplemental file 1 — Supplemental material. Download spectrum.04540-22-s0001.pdf, PDF file, 0.2 MB [file spectrum.04540-22-s0001.pdf]

Spectrum04540-22. PET-CT and MRI assessments in a mouse model of implant-related bone and joint *Staphylococcus aureus* infection

Supplemental Material

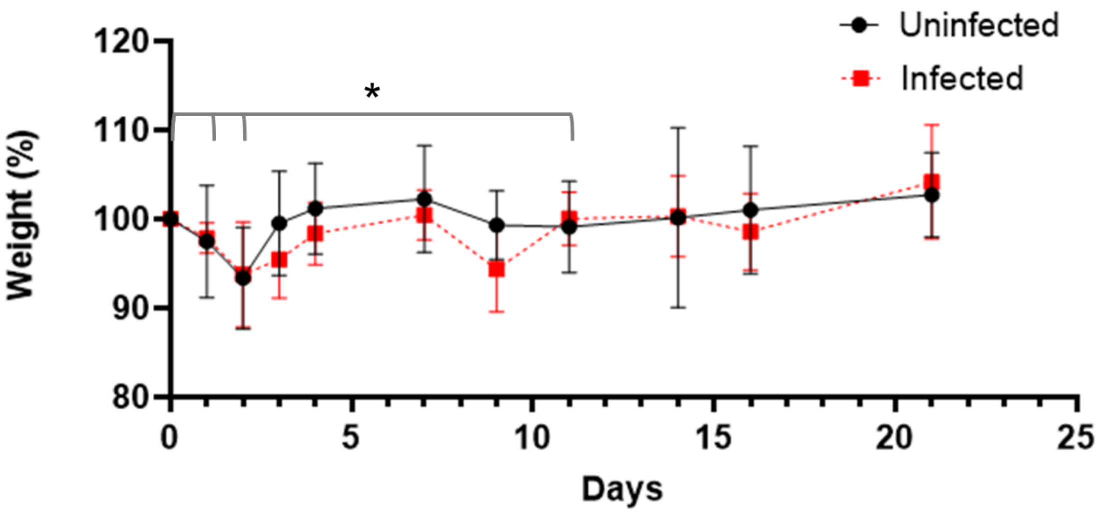

Figure S1. Body weight progression. Both groups experienced significant weight loss at 48 h post-intervention. The uninfected group recovered at 72 h, and the infected g did not completely recover for almost 10 days *p<0.05 in the infected group, compared to the 48-h value.

for almost 10 days *p<0.05 in the infected group, compared to the 48-h value.

Table S1. Clinical signs noted each day after implantation in infected (n=10) and uninfected (n=6) mice

Day	Lameness Infected/uninfected	Piloerection Infected/uninfected
**D1**	60/50	30/0
**D2**	60/50	40/0
**D3**	80/50	60/0*
**D4**	80/50	40/0
**D7**	100/33*	20/17
**D9**	80/33	50/0
**D11**	80/33	50/0
**D14**	80/33	40/0
**D16**	80/17*	40/0
**D21**	60/17	40/33

Values are the percentages per group; *p<0.05 between groups
